# Case Report: Advanced breast invasive ductal carcinoma with erysipeloid cutaneous metastasis misdiagnosed as erysipelas

**DOI:** 10.3389/fonc.2025.1535421

**Published:** 2025-07-14

**Authors:** Weiju Gu, Jing Yuan, Mengting Dong, Jiayu Sheng, Ke Jiang

**Affiliations:** Department of Breast Diseases, Yueyang Hospital of Integrated Traditional Chinese and Western Medicine, Shanghai University of Traditional Chinese Medicine, Shanghai, China

**Keywords:** breast carcinoma, cutaneous metastasis, erysipeloid, pathology, immunohistochemistry

## Abstract

**Background:**

Breast cancer has become the second most common cancer after lung cancer. Patients may present with skin manifestations at the time of initial diagnosis, while erysipel-like carcinoma typically appears later, following initial treatment. This delay increases the risk of misdiagnosis.

**Case presentation:**

The patient was a 51-year-old female. A modified radical mastectomy for left breast carcinoma (pT2N3M0, stage IIIC; tumor size 4.6 cm × 4.5 cm × 1.6 cm, 14/21 axillary lymph nodes involved), HER2-positive type, was performed on April 21, 2021. In April 2024 (three years post-surgery), the patient developed unexplained redness and swelling in the skin of the left upper limb, accompanied by increased skin temperature. This was misdiagnosed as erysipelas of the upper limb. After one week of antibiotic treatment, the redness and swelling slightly subsided. In May 2024, the patient experienced dizziness and headaches without any obvious cause. Enhanced cranial MRI revealed multiple brain metastases, with possible lymph node metastasis in the left cervical region. The patient underwent whole-brain radiotherapy. During radiotherapy, erysipelas-like rashes developed on the left chest wall, upper limb, and right breast skin. In June 2024, a skin biopsy of the chest wall confirmed cutaneous metastasis. Following systemic anti-tumor treatment, both the skin and brain metastasis improved.

**Conclusion:**

Pathological biopsy should be emphasized when breast cancer patients develop localized rashes. Understanding the unique inflammatory manifestations of cutaneous metastasis is crucial for breast oncologists to enable early diagnosis, timely treatment, and improved overall survival.

## Introduction

Global cancer statistics for 2022 indicate that breast cancer has become the second most common cancer among women after lung cancer, with an annual incidence rate increase of 0.6%-1% (1, 2). It can metastasize to atypical sites such as the gastrointestinal tract, skin, and other organs (3, 4). The clinical manifestations of skin metastasis in breast cancer include red patches, purplish-red papules, small blisters, and asymptomatic hard nodules on the breast and adjacent skin. Among these, *carcinoma erysipeloides* (CE) accounts for only 3% of breast cancer skin metastases (5), and its appearance closely resembles *erysipelas*, making it easily misdiagnosed. Skin metastases may present with clinical features resembling *erysipeloid* rash, complicating diagnosis and increasing the risk of misdiagnosis. This paper reports a case of advanced invasive ductal carcinoma of the breast with dermatitis-like skin metastasis, aiming to provide insights and guidance for diagnosis and treatment. Informed consent was obtained from the patient.

## Case report

**Figure 1:**
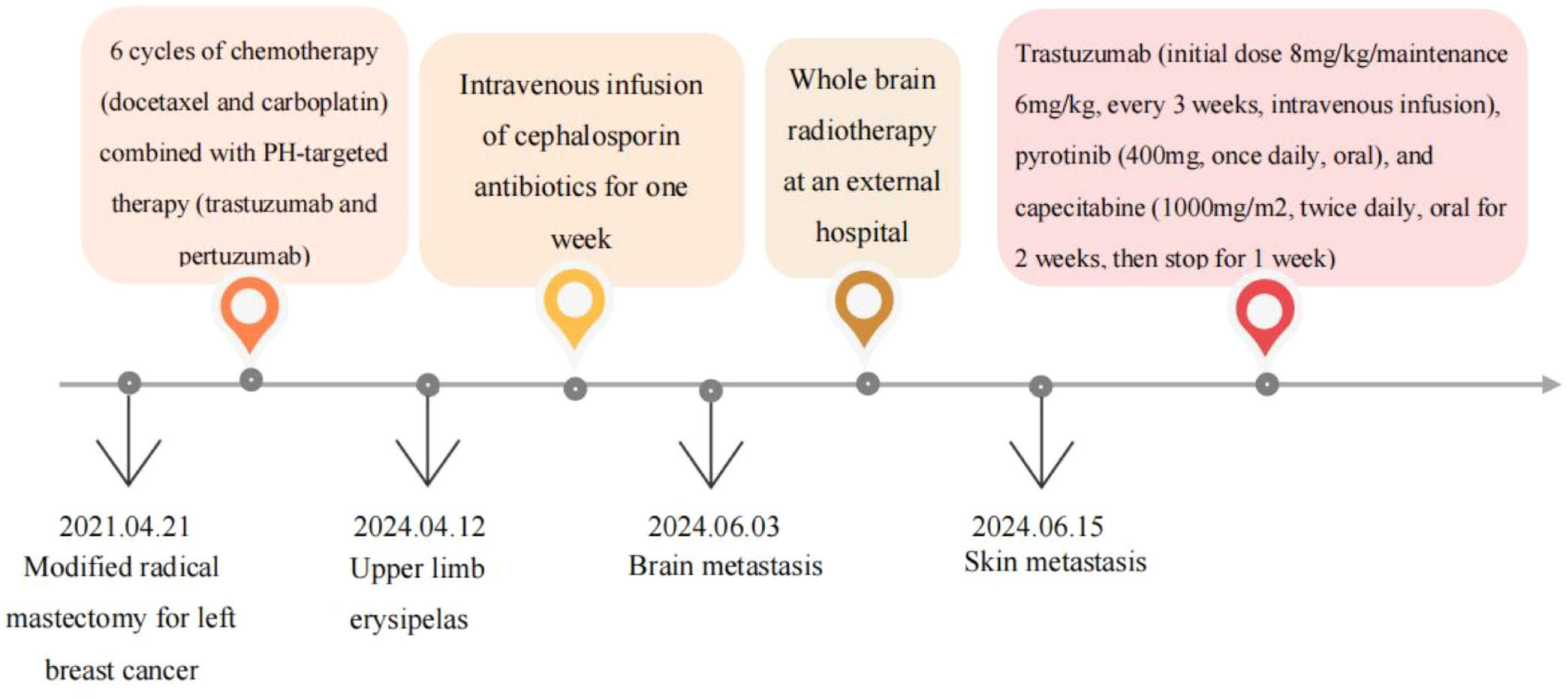
Timeline of treatment interventions.

We received a referral for a 51-year-old female patient who underwent a modified radical mastectomy for left breast cancer (pT2N3M0, stage IIIC, HER-2 positive) on April 21, 2021, after being treated at another hospital three years earlier. The overall treatment process is shown in the following diagram (**Figure 1**). Postoperative pathology revealed invasive ductal carcinoma of the left breast, grade II, with a tumor size of 4.6 cm × 4.5 cm × 1.6 cm, vascular invasion (+), no definite nerve invasion, and lymphoplasmacytic cellular infiltration between cancerous tissues (+), and left breast carcinoma (pT2N3MO,stage IIIC; tumor size 4.6cm × 4.5cm × 1.6cm, 14/21 axillary lymph nodes involved). Immunohistochemistry showed: Estrogen Receptor (ER) (-), Progesterone Receptor (PR) (-), Human Epidermal Growth Factor Receptor2 (HER2) (3+), MKI67 Protein (Ki67) (30%+), E-cadherin (E-cad) (+), Cell Counting Kit-8 (CK8) (+). She subsequently underwent six rounds of chemotherapy (docetaxel and carboplatin) combined with PH-targeted therapy (trastuzumab and pertuzumab). After adjuvant therapy, she received regular follow-ups every 3–6 months, during which the disease remained stable.

**Figure 2:**
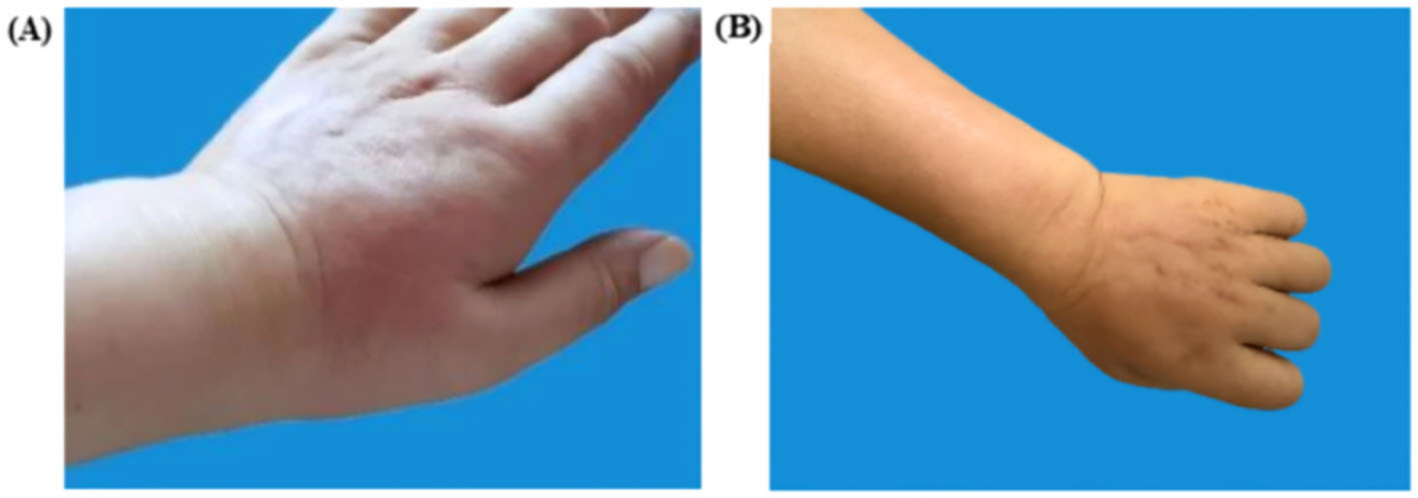
Left upper extremity lesions. **(A)** Pre-treatment erythema (April 12, 2024, the left dorsum of the hand shows redness with localized swelling and clear borders). **(B)** Post-treatment resolution (April 18, 2024, localized skin redness and swelling subsided after one week of antibiotic treatment).

On April 12, 2024, the patient’s arm on the side where the axillary clearance was performed—especially the back of the left hand—developed erythema and edema (**Figure 2**). The patient’s general condition was stable, with no obvious fever or chills. A blood test showed a white blood cell count of 9.6 × 10^9/L. She visited the dermatology department and was diagnosed with erysipelas of the upper limb. After receiving a week-long course of intravenous cephalosporin antibiotics, the localized redness and swelling in the left upper limb gradually subsided (**Figure 2**).

**Figure 3:**
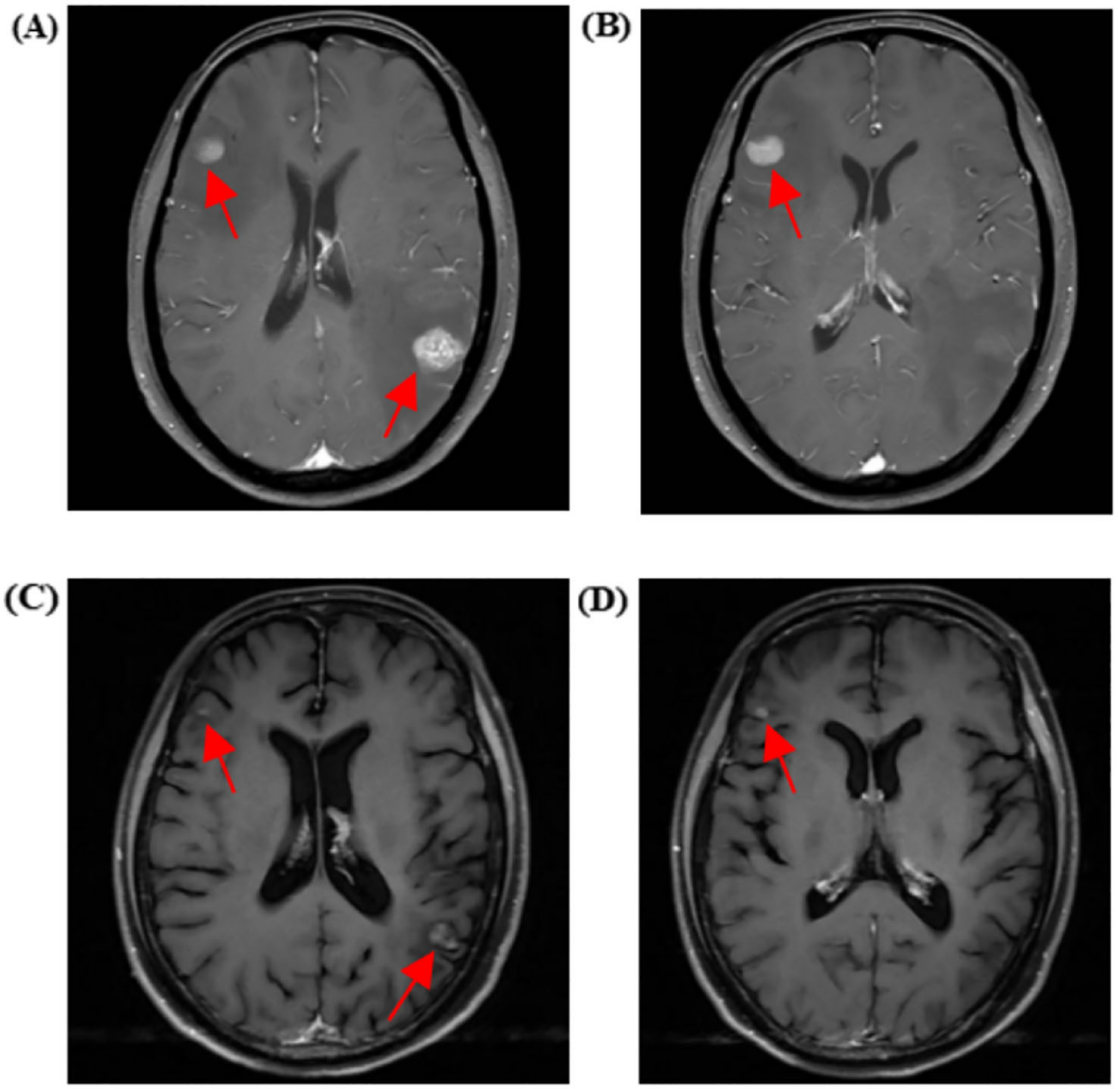
Cranial MRI showing metastatic lesions before and after treatment. **(A, B)** Cranial enhanced MRI on June 3, 2024, reveals metastases in the right frontal cortex, left parietal cortex, and left cerebellar hemisphere (the largest measuring approximately 11×13 mm). **(C, D)** Cranial-enhanced Magnetic Resonance Imaging (MRI) performed on July 30, 2024, shows that after whole-brain radiotherapy and antitumor treatment, the metastatic lesions in the right frontal lobe cortex, left parietal lobe cortex, and left cerebellar hemisphere (largest approximately 6*8mm) had reduced in size by 46% compared to before.

A month later, the patient experienced dizziness, headache, nausea, and vomiting without apparent cause. Tumor markers indicated a significant increase in carcinoembryonic antigen (CEA) and carbohydrate antigen 125 (CA125) compared to previous levels. Enhanced cranial MRI suggested metastatic tumors in the right frontal lobe cortex, left parietal lobe cortex, and left cerebellar hemisphere, with the largest measuring approximately 11×13 mm (**Figure 3**). Whole-brain radiotherapy was performed at another hospital on June 5, 2024.

**Figure 4:**
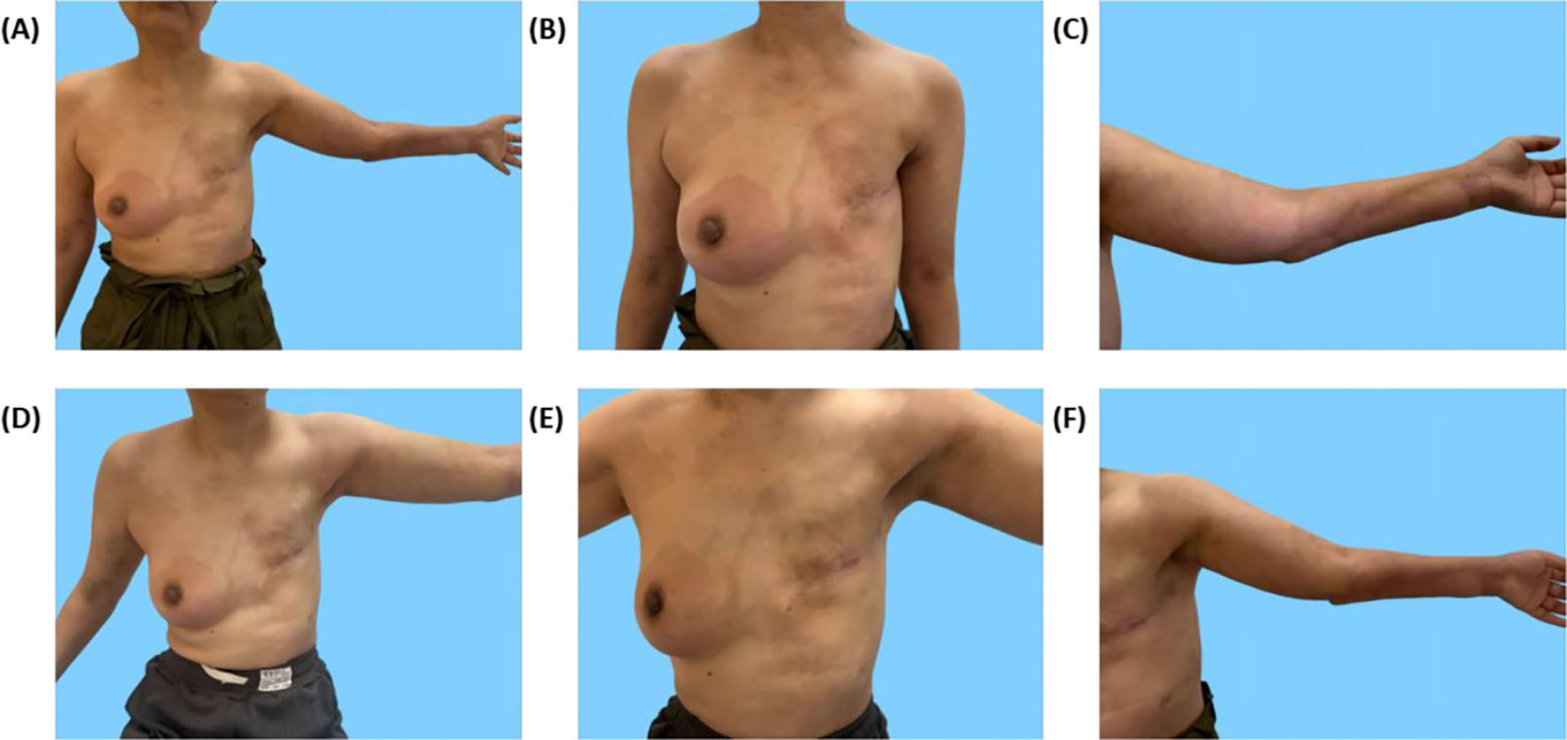
Skin lesions on the chest wall and left upper extremity before and after treatment. **(A–C)** Redness and swelling on the chest wall and left upper extremity with clear borders. **(D–F)** Reduction in erythema and regression of skin lesions after more than one month of systemic therapy.

During radiotherapy, the patient’s left chest wall skin developed redness and itching, and the area of skin lesions gradually spread to the right chest wall over time. The skin lesion on the left upper limb also reappeared (**Figure 4**). Breast MRI enhancement suggested multiple non-mass enhancements in the right breast, involving the nipple and skin. Positron Emission Computed Tomography/Computed Tomography (PET/CT) showed multiple low-density lesions in the brain with increased Fluoro Deoxy Glucose (FDG) metabolism, as well as left cervical lymph nodes with increased FDG metabolism, suggesting multiple brain metastases and left cervical lymph node metastases. No abnormal FDG metabolism was observed in other organs (lungs, liver, bones, uterus, gallbladder, etc.).

**Figure 5:**
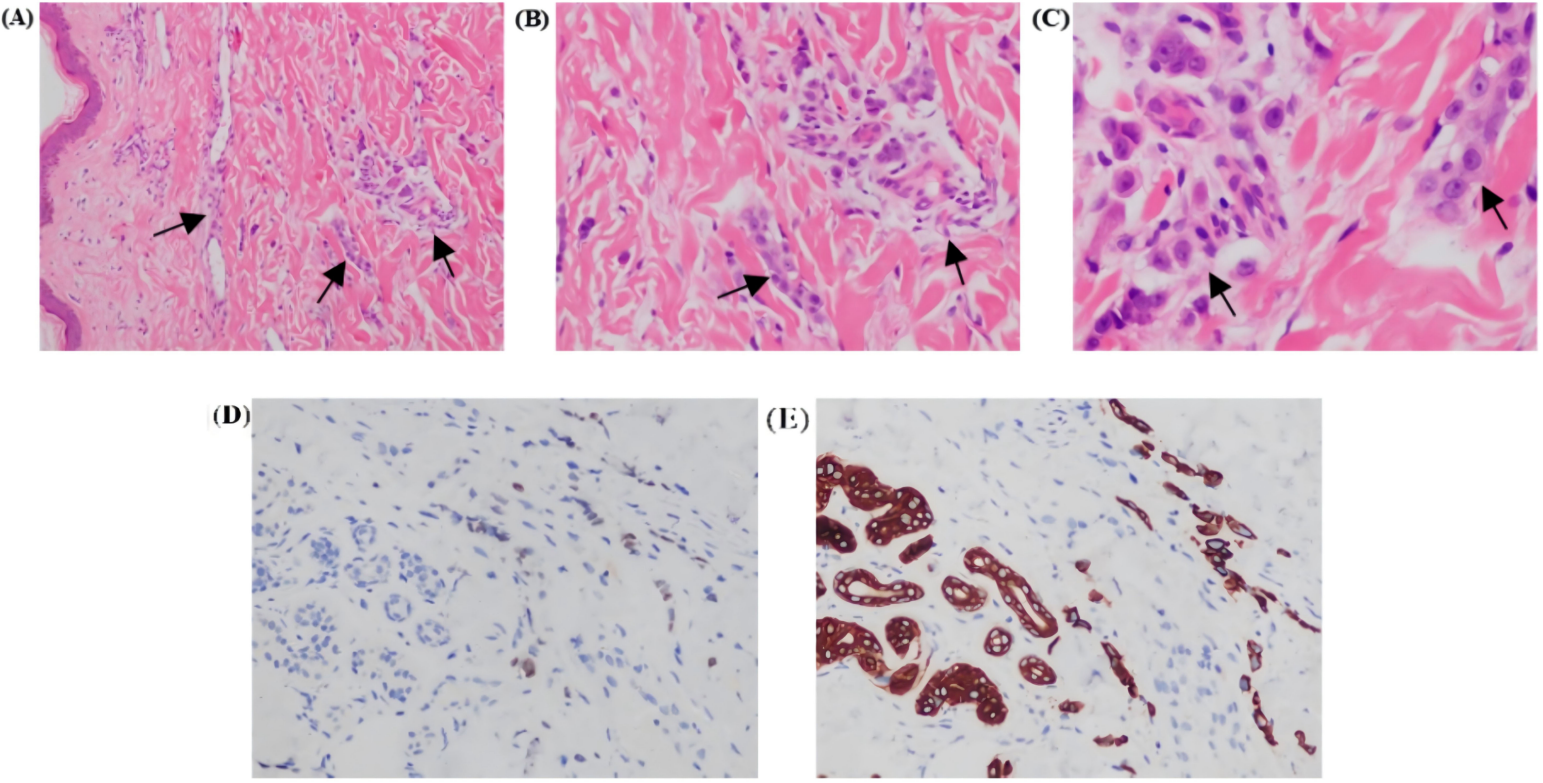
Photomicrographs of Hematoxylin and Eosin (H&E) stained sections revealed tumor cells arranged in cords and nests within the dermis **(A, B)**; H&E, (10x and 20×). Tumor cells separated by dermal collagen fiber bundles show marked atypia [**(C)**; H&E, 40×]. Tumor cells showed partial GATA-3 positivity [**(D)**; 20×]. Broad spectrum CK-20 immunopositivity seen in tumor cells [**(E)**; 20x]. All figures are provided in high-resolution TIFF format (300 dpi).

On June 15, 2024, a chest wall skin biopsy was performed. Histopathology showed infiltrating carcinoma consistent with breast cancer metastasis. Immunohistochemistry were as follows: ER (-), PR (-), HER2 (3+), Ki67 (70%+), GATA binding protein 3(GATA-3)(partially ±), Broad-spectrum Cytokeratin (CK wide) (+), CK5/6 (-), E-cad (-), and TP53 tumor protein (p53) (-) (**Figures 4**, **5**). At this point, the patient was diagnosed with multiple metastases of malignant tumors in the left breast, brain, lymph nodes, and skin—HER2-positive type. On June 30, 2024, she began treatment with inetetamab trastuzumab (first dose 8 mg/kg/, maintenance dose 6mg/kg, once every 3 weeks, intravenous infusion), pyrotinib (400mg, once daily, oral), and capecitabine (1000mg/m², twice daily, oral for 2 weeks followed by 1 week off). After one cycle of treatment with this regimen, the patient’s skin metastatic lesions showed regression, with large areas of infiltrative erythema darkening and shrinking in size. Tumor markers CEA and CA125 also decreased. A follow-up enhanced MRI of the head indicated that multiple metastatic lesions had shrunk, with the largest lesion reduced to approximately 6*8mm—representing a 46% regression, assessed as partial response (PR). As of the last follow-up in September 2024, the patient’s condition remains stable.

## Discussion

Skin metastasis is a type of secondary cancer in which malignant cells spread from the primary tumor to the skin through blood circulation or lymphatic vessels. Its incidence is approximately 0.6%-9% (6), typically occurring in the later stages of cancer. About 61.9% of patients with skin metastasis also present with one or more visceral organ metastases (7), and the median survival rate is only 6–8 months (8). In this case, the patient was diagnosed alongside extensive brain metastasis including rapid disease progression. Therefore, early identification of skin metastasis is critical to securing valuable time for systemic anti-tumor treatment.

Skin metastases can originate from a wide range of primary tumors, including those of the lungs, breasts, gastrointestinal tract, kidneys, liver, prostate, bladder, uterus, and ovaries (9). Regardless of the origin, the most common histological subtype of the primary tumor associated with skin metastasis is adenocarcinoma (10). Adenocarcinoma appears more likely to metastasize to the skin compared to other histological types, though the mechanism remains unclear. Some scholars speculate (11) that this may be related to the affinity of adenocarcinoma for dermal lymphatic vessels, which allows them to adhere to the lymphatic endothelial cells, form clusters, block lymphatic flow, and evade immune surveillance by lymphocytes.

Compared with lung cancer—which more commonly spreads to the trunk, scalp, and limbs—breast cancer skin metastasis is most commonly found on the trunk (10). Approximately 84.8% of patients present with metastasis on the same side as primary breast cancer, and about 9.1% of patients have metastasis on both sides of the chest wall (8). In this case, skin metastasis initially appeared on the left limb and later involved both sides of the chest wall. A single patient may exhibit one or more clinical types of skin metastasis. Nodular carcinoma is the most common, accounting for approximately 80% of cases. Clinically, it often presents as painless, firm, solitary or multiple pale red or red nodules of the body surface. Pathologically, it shows cord-like or lump-like infiltration of tumor cells in the dermis layer (12, 13). Erysipelas-like *carcinoma* accounts for only about 3% of skin metastases. Clinically, it presents as red or erythematous, infiltrative, hard, and tough plaques, with low skin temperature. Pathologically, it features tumor cells in clusters or cords within the full thickness of the dermis and lymphatic vessels, with minimal or no inflammatory cell infiltration (14). Tumor cells can adhere to lymphatic endothelial cells through specific adhesion molecules, forming cell clusters that retrograde spread and invade lymphatic vessels extensively. The dermal and subcutaneous lymphatic pathways may then become filled with nest-like and cord-like polymorphic tumor cells leading to lymphatic obstruction, capillary congestion, and lymphedema. In some patients, localized skin temperature may be elevated, further mimicking *erysipelas* and making it easy to misdiagnose. In this case, the patient was initially misdiagnosed with simple *erysipelas* of the left upper limb. Several factors contributed to this error. First, the clinical manifestations of erysipelas-like cancer are similar to those of simple *erysipelas*—both presenting with redness and swelling. Second, the condition occurred during summer, a season when lymphatic edema is more common in post-surgical breast cancer patients. The fact that the lesion appeared on the previously affected side added to the confusion. Third, the patient had an elevated white blood cell count. Finally, there was clear positive feedback on the antibiotics, as the redness and swelling subsided temporarily.

Clinical observations indicate that for patients with inflammatory skin lesions who initially respond to treatment (15), particularly those with a history of malignant tumors, it is crucial to maintain a high degree of vigilance regarding the potential metastasis of the primary tumor (10, 16–18). In cases of suspected *erysipelas*, performing local skin pathology and immunohistochemistry may risk aggravating the lesions or triggering systemic infection. Therefore, some scholars have suggested the use of dermoscopy before invasive procedures such as biopsy and immunohistochemistry (19, 20). If polymorphic vascular structures appear in the skin tissue under a microscope, this should prompt further histopathological and immunohistochemical investigation. Additionally, since skin metastasis is a manifestation of late-stage tumors and is often accompanied by multiple visceral metastases, a comprehensive systemic tumor evaluation should be conducted to avoid missing metastatic foci.

A study involving 27 patients with skin metastasis from breast cancer found that luminal-type breast cancer is the most common molecular subtype for skin metastasis, followed by HER2-positive and triple-negative breast cancer (21). The vast majority of skin metastases from breast cancer can achieve significant relief after systemic anti-tumor therapy combined with local treatment. For patients presenting with isolated skin metastases, local treatments include traditional surgery and radiotherapy, as well as newer approaches such as photodynamic therapy, direct injection of antitumor agents into the tumor, and application of antitumor agents on the surface of skin lesions, which have been continuously developed and refined in recent years. However, in cases where skin metastasis is found along with other visceral metastases, systemic treatment should still be the primary therapeutic approach, with local treatment as an adjunct. Treatment planning should follow the principles for advanced breast cancer. In this case, the patient was diagnosed with HER2-positive advanced breast cancer with skin, brain, and lymphatic metastasis. After the whole-brain radiotherapy, a systemic treatment regimen combining small molecule Tyrosine Kinase Inhibitor (TKI), chemotherapy, and large-molecule monoclonal antibodies. Following this treatment, both skin and brain metastases showed measurable improvement, indicating that the anti-tumor therapy was effective.

## Conclusion

Skin is a relatively rare site for metastasis in breast cancer and other primary tumors. During diagnosis and treatment, cutaneous metastases are often misdiagnosed due to its clinical similarity to skin conditions such as erysipelas-like rash—especially when antibiotic treatment appears effective. To improve diagnostic accuracy breast surgeons should collaborate with dermatopathologists, radiologists, and oncologists. This is just a single case report, and a relatively short follow-up period. Therefore, we need more cases and cohort studies with longer follow-up periods to support these observations. Future multi-center studies with extended follow-up periods are warranted to validate these findings.

## Data Availability

The original contributions presented in the study are included in the article/supplementary material. Further inquiries can be directed to the corresponding authors.

## References

[1] Siegel RL Miller KD Fuchs HE Jemal A . Cancer statistics. CA Cancer J Clin. (2022) 72:7–33. doi: 10.3322/caac.21708, PMID: 35020204

[2] Giaquinto AN Sung H Newman LA Freedman RA Smith RA Star J . Breast cancer statistics. CA Cancer J Clin. (2024) 74:477–95. doi: 10.3322/caac.21863, PMID: 39352042

[3] Dayan D Lukac S Rack B Ebner F Fink V Leinert E . Effect of histological breast cancer subtypes invasivelobular versus non-special type on survival in early intermediate-to-high-risk breast carcinoma: results from the SUCCESS trials. Breast Cancer Res. (2023) 25:153. doi: 10.1186/s13058-023-01750-0, PMID: 38098086 PMC10722735

[4] Dilawar H Ahmed A Habib S Iqbal J Abdul Rehman T Hadi I . Gastric metastasis from invasive lobular breast cancer, resembling primary gastric cancer. J Nucl Med Technol. (2024) 52:68–70. doi: 10.2967/jnmt.123.266035, PMID: 37699646

[5] Rehman S Naveed MA . Skin metastasis in breast cancer patients; a case series. J Cancer Allied Spec. (2020) 6:307. doi: 10.37029/jcas.v6i1.307, PMID: 37197144 PMC10187598

[6] Lian Z Zhifang Z Hongzhi G Xin W Juan W . Clinical and histopathological analysis of 62 cases of cutaneous metastatic carcinoma. Chin J Lepr Skin Dis. (2022) 38:803–6. doi: 10.12144/zgmfskin202211803

[7] Jaros J Hunt S Mose E Lai O Tsoukas M . Cutaneous metastases: A great imitator. Clin Dermatol. (2020) 38:216–22. doi: 10.1016/j.clindermatol.2019.10.004, PMID: 32513401

[8] Shin DM Jung YJ Kim H Oh SJ Shim J Lee JH . Clinical characteristics and survival analysis of cutaneous metastases in a single tertiary centre in Korea. J Eur Acad Dermatol Venereol. (2023) 37:2311–8. doi: 10.1111/jdv.19361, PMID: 37467154

[9] Sivakanthan T Tanner J Mahata B Agrawal A . Investigating the role of tumour-to-skin proximity in predicting nodal metastasis in breast cancer. Breast Cancer Res Treat. (2024) 205:109–16. doi: 10.1007/s10549-023-07230-5, PMID: 38308767 PMC11063104

[10] Eroğlu İ Üner A Gürler F Yazıcı O Özet A Özdemir N . Carcinoma erysipeloides, a case-report and review of the sixty-nine cases in the literature. Am J Med Sci. (2025) 369:485–90. doi: 10.1016/j.amjms.2024.10.008, PMID: 39454728

[11] Wenlin W He Z Zhenfeng H Zhikuan X Rongyan Y . Clinical and literature analysis of erysipelas skin metastases. J Pract Dermatol. (2020) 13:201–4. doi: 10.11786/sypfbxzz.1674-1293.20200403

[12] Gonzálezmartínez S Pizarro D Pérezmies B Caniegocasas T Curigliano G Cortés J . Clinical, pathological, and molecular features of breast carcinoma cutaneous metastasis. Cancers Basel. (2021) 13:5416. doi: 10.3390/cancers13215416, PMID: 34771579 PMC8582578

[13] Mulvaney PM Schmults CD . Molecular prediction of metastasis in cutaneous squamous cell carcinoma. Curr Opin Oncol. (2020) 32:129–36. doi: 10.1097/CCO.0000000000000609, PMID: 31850970

[14] Xiaobin L Bin W . Analysis of the diagnostic process of a case of breast cancer with skin metastasis misdiagnosed as sebaceous gland cyst with infection. Chin Gen Pract. (2020) 23:2604–6. doi: 10.12114/j.issn.1007-9572.2020.00.164

[15] Junak M Jecius H Erdrich J . Cutaneous metastasis in the setting of both colon and breast primary Malignancies. Case Rep Gastrointest Med. (2020) 2020:8852459. doi: 10.1155/2020/8852459, PMID: 33062353 PMC7542512

[16] Tan WF Voo SYM . A sinister rash in a lady with breast Malignancy. Med J Malaysia. (2021) 76:275–7. Available at: https://openurl.ebsco.com/contentitem/gcd:149427828?sid=ebsco:plink:crawler&id=ebsco:gcd:149427828, PMID: 33742647

[17] Pliakou E Lampropoulou DI Nasi D Aravantinos G . Skin metastases from gastric cancer, a rare entity masquerading as erysipelas: A case report. Mol Clin Oncol. (2022) 16:110. doi: 10.3892/mco.2022.2543, PMID: 35620210 PMC9112402

[18] Kurmus GI Canpolat F Gönül M Gökçe A Kartal SP . Cutaneous metastases of signet-ring cell gastric carcinoma: A case report. Curr Health Sci J. (2024) 50:444–7. doi: 10.12865/CHSJ.50.03.12, PMID: 39574817 PMC11578364

[19] Alexis F Leggett LR Agarwal N Bakhtin Z Farabi B . Carcinoma erysipeloides with clinical and dermatoscopic features: an overlooked clinical manifestation of breast cancer. Cureus. (2022) 14:e23445. doi: 10.7759/cureus.23445, PMID: 35494933 PMC9038511

[20] Tiodorovic D Stojkovic-Filipovic J Marghoob A Argenziano G Puig S Malvehy J . Dermatoscopic patterns of cutaneous metastases: A multicentre cross-sectional study of the International Dermoscopy Society. J Eur Acad Dermatol Venereol. (2024) 38:1432–8. doi: 10.1111/jdv.19962, PMID: 38483241 PMC11630489

[21] Xinmiao Y Lin D Yan X Yongqiang Z . Characteristics and prognosis of skin metastasis of female breast cancer. Chin J Cancer Prev Treat. (2021) 28:1329–1332 + 1338. doi: 10.16073/j.cnki.cjcpt.2021.17.09

